# Lung cancer in lifetime nonsmoking men – results of a case-control study in Germany

**DOI:** 10.1054/bjoc.2000.1518

**Published:** 2001-01-01

**Authors:** M Kreuzer, M Gerken, L Kreienbrock, J Wellmann, H E Wichmann

**Affiliations:** BfS – Federal Office of Radiation Protection, Institute of Radiation Hygiene, Ingolstaedter Landstrasse 1, Neuherberg, 85764; GSF – National Research Center for Environment and Health, Institute of Epidemiology, Ingolstaedter Landstrasse 1, Neuherberg, 85764; Hannover School of Veterinary Medicine, Institute for Biometry, Epidemiology and Information Processing, Buenteweg 2, Hannover, 30559; University of Münster, Institute of Epidemiology and Social Medicine, Domagkstrasse 3, Münster, 48129, Germany

**Keywords:** lung cancer, case-control study, radon, nonsmokers

## Abstract

Epidemiological studies of lung cancer among nonsmoking men are few. This case–control study was conducted among lifetime nonsmoking men between 1990 and 1996 in Germany to examine lung cancer risk in relation to occupation, environmental tobacco smoke, residential radon, family history of cancer and previous lung disease. A total of 58 male cases with confirmed primary lung cancer and 803 male population controls who had never smoked more than 400 cigarettes in their lifetime were personally interviewed by a standardized questionnaire. In addition, 1-year radon measurements in the living and bedroom of the subjects' last dwelling were carried out. Unconditional logistic regression was used to calculate odds ratios (OR) and 95% confidence intervals (CI). Having ever worked in a job with known lung carcinogens was associated with a two-fold significantly increased lung cancer risk (OR = 2.2; Cl = 1.0–5.0), adjusted for age and region. The linear trend test for lung-cancer risk associated with radon exposure was close to statistical significance, demonstrating an excess relative risk for an increase in exposure of 100 Bq m^−3^ of 0.43 (*P* = 0.052). Nonsignificantly elevated effects of exposure to environmental tobacco smoke in public transportation and in social settings were observed. No associations with a family history of cancer or previous lung diseases were found. Our results indicate that occupational carcinogens and indoor radon may play a role in some lung cancers in nonsmoking men. © 2001 Cancer Research Campaign http://www.bjcancer.com


British Journal of Cancer (2001) 84(1), 134–140
© 2001 Cancer Research Campaign
doi: 10.1054/ bjoc.2000.1518, available online at http://www.idealibrary.com on IDEAL®

http://www.bjcancer.com

### Lung cancer in lifetime nonsmoking men – results of a case-control study in Germany

**M Kreuzer[1,2], M Gerken[2], L Kreienbrock[2,3], J Wellmann[2,4] and HE Wichmann[2]**

[1]BfS – Federal Office of Radiation Protection, Institute of Radiation Hygiene, Ingolstaedter Landstrasse 1, 85764 Neuherberg; [2]GSF – National Research Center for Environment and Health, Institute of Epidemiology, Ingolstaedter Landstrasse 1, 85764 Neuherberg; [3]Hannover School of Veterinary Medicine, Institute for Biometry, Epidemiology and Information Processing, Buenteweg 2, 30559 Hannover; [4]University of Münster, Institute of Epidemiology and Social Medicine, Domagkstrasse 3, 48129 Münster, Germany

**Summary** Epidemiological studies of lung cancer among nonsmoking men are few. This case–control study was conducted among lifetime nonsmoking men between 1990 and 1996 in Germany to examine lung cancer risk in relation to occupation, environmental tobacco smoke, residential radon, family history of cancer and previous lung disease. A total of 58 male cases with confirmed primary lung cancer and 803 male population controls who had never smoked more than 400 cigarettes in their lifetime were personally interviewed by a standardized questionnaire. In addition, 1-year radon measurements in the living and bedroom of the subjects' last dwelling were carried out. Unconditional logistic regression was used to calculate odds ratios (OR) and 95% confidence intervals (CI). Having ever worked in a job with known lung carcinogens was associated with a two-fold significantly increased lung cancer risk (OR = 2.2; CI = 1.0–5.0), adjusted for age and region. The linear trend test for lung-cancer risk associated with radon exposure was close to statistical significance, demonstrating an excess relative risk for an increase in exposure of 100 Bq $m^{-3}$ of 0.43 ($P$ = 0.052). Nonsignificantly elevated effects of exposure to environmental tobacco smoke in public transportation and in social settings were observed. No associations with a family history of cancer or previous lung diseases were found. Our results indicate that occupational carcinogens and indoor radon may play a role in some lung cancers in nonsmoking men. © 2001 Cancer Research Campaign http://www.bjcancer.com

**Keywords**: lung cancer; case-control study; radon; nonsmokers

Tobacco smoke is the major risk factor for lung cancer and accounts for about 80–95% of lung cancers in men (Parkin et al, 1994). Many of the potential risk factors for lung cancer other than active smoking, such as environmental tobacco smoke (Hackshaw et al, 1997; Boffetta et al, 1998), residential radon (BEIR VI, 1998), diet (Brennan et al, 2000), occupational hazards (Keller and Howe, 1993; Pohlabeln et al, 2000), previous non-neoplastic lung disease (Wu et al, 1995; Mayne et al, 1999), family history of lung cancer (Schwartz et al, 1996; Brownson et al, 1997) and genetic susceptibility (Bennett et al, 1999) show relative risk estimates less than two, which are about ten times smaller in magnitude than the relative risk of cigarette smoking. To determine the extent to which these risk factors contribute to lung cancer, studies of lifetime nonsmokers are preferable, because otherwise adequate controlling for smoking is difficult.

About 10–30% of European and American women with lung cancer report never having smoked in their life, in contrast to only 2% of male cases (Koo and Ho, 1990). A large body of evidence on potential risk factors for lung cancer in nonsmokers has been accumulated (Brownson et al, 1998); this knowledge, however, is nearly exclusively based on studies conducted in women. We therefore used data from a large case–control study on lung cancer and indoor radon, conducted from 1990–1996 in Germany, to focus on nonsmoking men. Some of the findings have been published previously (Boffetta et al, 1998; Kreuzer et al, 1998, 1999, 2000; Brüske-Hohlfeld et al, 2000; Kreienbrock et al, 2000). The present paper describes our major findings on the relation between residential radon, occupational exposure, environmental tobacco smoke, family history of cancer and previous lung disease and risk of lung cancer in nonsmoking men.

*Received 8 June 2000*
*Revised 31 August 2000*
*Accepted 4 September 2000*

*Correspondence to:* M Kreuzer

### MATERIALS AND METHODS

Data on nonsmokers were derived from a case–control study of lung cancer and indoor radon conducted from 1990–1996 in several regions of East and West Germany. The data collection methods have been described previously (Kreuzer et al, 2000; Kreienbrock et al, 2000). In brief, newly diagnosed cases with histologically or cytologically confirmed lung cancer as a primary tumour were recruited from 15 study clinics in the defined study area. Cases were eligible if: (1) they were less than 76 years old; (2) they were currently resident in the study area; (3) they had lived in Germany for more than 25 years; (4) the interviews were within 3 months after diagnosis; and (5) they were not too ill. The response rate of eligible cases was 76%. Population controls satisfying inclusion criteria 1–3 were randomly selected from mandatory registries or by modified random-digit dialing and were frequency matched to the cases on sex, age and region. The response rate of eligible controls was 41%.

A total of 4303 cases and 4451 population controls including smokers and nonsmokers, as well as men and women, were personally interviewed by a trained interviewer. A standardized questionnaire was used to determine detailed residential history, active and passive smoking history, occupational exposure, dietary habits, previous lung disease and a family history of cancer. One-year measurements of radon concentrations in the living and bedroom of the subjects' last dwellings were obtained by α-track detectors. The present analyses were restricted to nonsmoking men. In accordance with previously published definitions (Boffetta et al, 1998; Kreuzer et al, 2000), subjects who had never smoked more than 400 cigarettes in their life were defined as lifetime nonsmokers.

The lifelong occupational history was recorded including job title, branch and industry as well as the dates of all jobs held for at least 6 months. Job titles were coded according to the German standard classifications provided by the Federal Office of Statistics (Statistisches Bundesamt, 1975). A list of jobs, branches and industries in which a risk for lung cancer has been confirmed (A-list), or suspected (B-list), was applied to the job title and industry codes, as recently published by Ahrens and Merletti (1998). Table 2 lists in detail all occupations and industries classified as belonging to the A-list. A subject was defined as exposed in an A-list (B-list) job if he had worked for at least 6 months in an occupation on A-list (B-list). Cumulative duration of exposure in years in jobs of list A (or both lists combined) was calculated and categorized as less than 10 years or 10 or more years.

Residential radon exposure was quantified as the time-weighted average of the radon concentrations in the living room and bedroom of the present home, taking into account the individual's time proportion spent in both rooms (Kreienbrock et al, 2000). Changes due to reconstruction of the house (e.g. windows) over the years were considered by using correction factors developed in a multivariate model (Gerken et al, 2000). The questionnaire on environmental tobacco smoke (ETS) gathered information on ETS exposure during adulthood at home (spouse or other cohabitants), at the workplace, in vehicles and at other public places (social settings). The results on lung-cancer risk and ETS for nonsmoking women and men combined have recently been published (Kreuzer et al, 2000). In accordance with this analysis we used the cumulative duration of exposure in hours weighted for a subjective index for level of smokiness as the variable for exposures to ETS. Subjects who were never exposed comprised the reference category and exposures above (below) the 75th percentile were considered as high (low) for each source of exposure.

All subjects were asked if they had ever been told by a physician that they had asthma, tuberculosis, emphysema, chronic bronchitis or pneumonia at any age at least 2 years before diagnosis of cancer (or date of interview for controls). Information on history of cancer among first-degree relatives (parents, siblings and offspring) was gathered, including age at disease, site of cancer and relation to the subject. Subjects were defined as having cancer in the family if at least one relative with cancer was reported. This factor was defined for lung cancer and cancer of any site. Information on smoking habits of the relatives was available for parents only. A food frequency questionnaire was used to gather dietary habits.

Odds ratios and 95% confidence intervals were calculated from unconditional logistic regression models (Breslow and Day, 1980). All odds ratios were adjusted for age and region (three areas). We examined the effects of each of the above-mentioned risk factors. Potential confounders for these factors such as occupational exposure (ever/never exposed to a job of A-list), residential radon (continuous variable), environmental tobacco exposure outside from home (workplace, transportation and social settings), pre-existing lung disease (history of at least one lung condition) and diet (daily vs less-than-daily consumption of fresh fruits) were added to the models. Since no major confounding was found, results were presented adjusted for age and region only. According to previous analyses, radon exposure was categorized into < 50, 50–79, 80–139 and ≥ 140 Bq $m^{-3}$ (Kreienbrock et al, 2000). A linear trend test was performed by treating radon exposure as a continuous variate and calculating the excess relative risk (ERR) per additional exposure of 100 Bq $m^{-3}$. Potential differences in lifestyle and exposure to carcinogenic risk factors for lung cancer between subjects from East and West Germany were examined by repeating all analyses separately for each study area and discussing differences if present.

### RESULTS

A total of 58 male nonsmoking lung cancer patients and 803 male nonsmoking control subjects were studied. Table 1 shows their sociodemographic characteristics. The distribution of age was very similar among case and control subjects; mean ages being 57 and 59, respectively. Case and control subjects were broadly similar regarding marital status and educational levels, 35% of case and 42% of control subjects having completed at least 10 years of formal education. The predominant cell type was adenocarcinoma (59%), followed by squamous cell carcinoma (19%), small cell lung cancer (16%) and other types (7%).

Table 2 shows the number of cases and controls who had worked at some time for at least 6 months in industries and occupations entailing known carcinogens, showing that about 14% of case and 7% of control subjects had ever done so. The most frequently reported jobs and industries in the A-list were painters, railroad manufacturing workers, asphalt workers, iron and steel foundry workers and roofers, and these were associated with a 2.2-fold increased lung-cancer risk (Table 3). There was an increasing risk of lung cancer with increasing duration of exposure. Working

**Table 1** Characteristics of the study population

| | Cases | | Controls | |
|---|---|---|---|---|
| | *n* | % | *n* | % |
| Totals | 58 | 100.0 | 803 | 100.0 |
| Age in years (%) | | | | |
| < 50 | 11 | 19.0 | 97 | 12.1 |
| 50–54 | 9 | 15.5 | 120 | 14.9 |
| 55–59 | 16 | 27.6 | 202 | 25.2 |
| 60–64 | 10 | 17.2 | 178 | 22.2 |
| 65–69 | 6 | 10.3 | 131 | 16.3 |
| 70–74 | 6 | 10.3 | 75 | 9.3 |
| Study area | | | | |
| Eastern Germany | 31 | 53.4 | 359 | 44.7 |
| Western Germany | 27 | 46.6 | 444 | 55.3 |
| Family status | | | | |
| Single | 2 | 3.2 | 25 | 3.0 |
| Married | 55 | 95.2 | 719 | 91.6 |
| Widowed/Divorced | 1 | 1.6 | 59 | 5.4 |
| Years of school attendance | | | | |
| < 9 years | 1 | 1.6 | 6 | 0.8 |
| 9 years | 37 | 62.9 | 463 | 57.7 |
| 10–11 years | 13 | 22.6 | 128 | 16.0 |
| ≥ 12 years | 7 | 12.9 | 205 | 25.5 |
| Histologic type | | | | |
| Small cell carcinoma | 9 | 15.5 | – | – |
| Squamous cell carcinoma | 11 | 19.0 | – | – |
| Adenocarcinoma | 34 | 58.6 | – | – |
| Other histologic types | 4 | 6.9 | – | – |

© 2001 Cancer Research Campaign 

**Table 2** Subjects who worked for at least 6 months in industries and occupations entailing known lung carcinogens (A-List)[a]

| Industry | Occupation/process | Cases (*n*) | Controls (*n*) |
|---|---|---|---|
| Agriculture | Vineyard workers using arsenic insecticides (before 1942) | – | – |
| Mining | Arsenic mining, uranium mining, iron-ore mining, etc. (ore-mining only) | 1 | 2 |
| Asbestos production | Insulated material production (asbestos cement products, pipes, sheeting, etc.) | – | 3 |
| Metals (iron and steel basic industries) | Iron and steel founding | 1 | 7 |
| Metals (non-ferrous, basic industries: smelting alloying, refining, rolling, drawing, casting) | Copper smelting, zinc smelters, cadmium alloy production, aluminium production, etc. | – | 3 |
| Metals | Pickling operations, chromeplating, electroplating, brazing, nickel-cadmium battery manufacturing | – | 4 |
| Shipbuilding, railroad equipment manufacturing | Shipyards and dockyards and railroad manufacture workers | 2 | 15 |
| Gas | Coke plant workers and gas production workers | – | – |
| Construction | Insulators and pipe coverers | 1 | – |
| | Roofers | – | 4 |
| | Asphalt workers | 1 | 9 |
| Construction, automotive or any industry | Painters | 2 | 14 |
| Totals | | 8 | 61[b] |

[a]Source: Ahrens and Merletti, 1998; [b]Five controls have worked in more than one job or industry of A-list.

**Table 3** Odds ratios for lung cancer and working at least 6 months in a job with known (A-list) or suspected (B-list) lung carcinogens

| | Cases (*n*) | Controls (*n*) | OR | 95% CI |
|---|---|---|---|---|
| Never A-list | 50 | 747 | 1.0 | Referent |
| A-list | 8 | 56 | 2.2 | 1.00–4.98 |
| Duration of exposure in years | | | | |
| < 10 years | 3 | 36 | 1.3 | 0.40–4.55 |
| ≤ 10 years | 5 | 20 | 3.7 | 1.33–10.4 |
| Never A- or B-list | 38 | 613 | 1.0 | Referent |
| B- never A-list | 12 | 134 | 1.4 | 0.71–2.79 |
| A-list | 8 | 56 | 2.4 | 1.06–5.43 |
| Duration of exposure in years | | | | |
| <10 years | 6 | 68 | 1.5 | 0.59–3.57 |
| ≥10 years | 14 | 122 | 1.8 | 0.96–3.43 |

OR = odds ratio adjusted for age and region.

**Table 4** Radon concentrations in the present dwellings in Bq m$^{-3}$, individual occupancy-weighted average of living and bedroom

| | Cases/Controls | | |
|---|---|---|---|
| Characteristics | Total | West | East |
| Subjects with measurements (*n*) | 49/760 | 22/418 | 27/342 |
| Subjects without measurements (*n*) | 9/43 | 5/26 | 4/17 |
| Mean (Bq m$^{-3}$) | 80/61 | 40/50 | 113/74 |
| Median (Bq m$^{-3}$) | 51/46 | 35/40 | 73/51 |
| Minimum (Bq m$^{-3}$) | 20/11 | 20/11 | 27/13 |
| Maximum (Bq m$^{-3}$) | 519/557 | 89/341 | 519/557 |

< 10 years or ≥ 10 years in jobs of A-list, as compared to never working in such a job, was associated with a 1.3-fold (95% CI = 0.4–4.6) or 3.7-fold (95% CI = 1.3–10.4) increased lung-cancer risk, respectively. Taking subjects who had never worked in a job with known or suspected carcinogens as the reference category (66% of case subjects and 76% of control subjects), those working in an A-list job but never in a B-list job exhibited a 2.4-fold risk. Other potential risk factors of lung cancer exerted only minor confounding effects on these occupational risks. Thus, the OR for ever/never exposure to jobs of A-list was modified to OR = 2.3 (95% CI = 1.04–5.3) after adjustment for radon exposure and to OR = 2.2 (95% CI = 0.96–4.82) after adjustment for educational level. Risk estimates were consistently much higher for subjects enrolled in the study area in West Germany than for those in East Germany, though numbers of exposed subjects in East Germany were small.

In Table 4 characteristics of the radon exposure in the present home are given. On average the current residence was occupied for 23 years in all groups, demonstrating overall a mean annual radon exposure of 80 Bq m$^{-3}$ among case subjects and 61 Bq m$^{-3}$ among control subjects. In cases with small cell carcinoma, the mean radon exposure was substantially higher (154 Bq m$^{-3}$) than among patients with non-small cell lung cancer (65 Bq m$^{-3}$). Due to the high uranium contents of the ground in the selected study areas of Eastern Germany (Thuringia and Saxony), control subjects of this area showed appreciably higher mean radon levels in their houses (74 Bq m$^{-3}$) than those of West Germany (50 Bq/m$^3$).

Overall the odds ratios for lung cancer and radon exposure using less than 50 Bq m$^{-3}$ as reference category were 1.3 (95% CI = 0.6–2.6), 1.5 (95% CI = 0.6–3.5) and 2.0 (95% CI = 0.7–5.8) for categories 50–79, 80–139 and 140+ Bq m$^{-3}$, respectively, and the estimated excess relative risk (ERR) per 100 Bq m$^{-3}$ was 0.43 ($P = 0.052$) (Table 5). Since there was a strong interaction for lung cancer risk with respect to radon exposure and study area, risk estimates were calculated for the eastern and western parts separately. Due to the low radon exposures in the western study area, cases and controls were predominantly in the reference category, and no elevated risk nor a significant trend was observed. However, the power to detect such an effect was low. In contrast to this, a clear significant excess relative risk of 60% per increase of 100 Bq m$^{-3}$ was found in the eastern study area. In comparison with radon concentrations up to 50 Bq m$^{-3}$, the odds ratio was 3.0 for 50–79 Bq m$^{-3}$, 3.3 for 80–139 Bq m$^{-3}$ and 5.0 at concentrations exceeding 140 Bq m$^{-3}$. Overall, odds ratios did not substantially change after adjustment for potential confounders, for example in the entire study region the ERR for an increase of 100 Bq m$^{-3}$ was 0.44 (CI = 0.01–1.05) after adjustment for occupation.

As shown in Table 6, the spouse or other cohabitants were negligible as potential sources of ETS exposure in adulthood at home,

*© 2001 Cancer Research Campaign*

**Table 5** Odds ratio for lung cancer and residential radon exposure (time-weighted average annual radon exposure in the last dwelling in Bq $m^{-3}$)

| | Cases | Controls | OR | 95% CI |
|---|---|---|---|---|
| Total | | | | |
| < 50 | 23 | 429 | 1.0 | Referent |
| 50–79 | 13 | 192 | 1.3 | 0.62–2.55 |
| 80–139 | 8 | 97 | 1.5 | 0.64–3.52 |
| > 140 | 5 | 42 | 2.0 | 0.72–5.80 |
| ERR for 100 Bq $m^{-3}$ | | | 0.43 | –0.004–1.05 |
| East | | | | |
| < 50 | 6 | 167 | 1.0 | Referent |
| 50–79 | 9 | 86 | 3.0 | 1.03–8.77 |
| 80–139 | 7 | 61 | 3.3 | 1.07–10.3 |
| > 140 | 5 | 28 | 5.0 | 1.43–17.6 |
| ERR for 100 Bq $m^{-3}$ | | | 0.60 | 0.11–1.30 |
| West | | | | |
| < 50 | 17 | 262 | 1.0 | Referent |
| 50–79 | 4 | 106 | 0.6 | 0.20–1.85 |
| 80–139 | 1 | 36 | 0.5 | 0.06–3.83 |
| > 140 | – | 14 | – | |
| ERR for 100 Bq $m^{-3}$ | | | –0.74 | –0.967–1.006 |

Nine case subjects and 43 control subjects were excluded due to missing radon values; OR = odds ratio adjusted for age and region.

since almost no such exposure was reported by nonsmoking men. In contrast, 75% of control subjects reported ETS exposure in public places such as workplace, transportation or restaurants. High ETS exposure outside the home was associated with elevated excess risks that did not achieve statistical significance. Thus, the odds ratio for ETS exposure in public transportation was 2.7 (CI = 0.9–8.0) and 1.5 (CI = 0.7–3.4) among subjects highly exposed to ETS at social settings, respectively. When all sources of ETS exposure outside the home were considered jointly, a slightly elevated exposure–response relationship was found. No influence of additional adjustment for occupational exposure, consumption of fresh fruits or radon exposure was observed.

No elevated risk was observed for such prior lung conditions as tuberculosis, asthma, chronic bronchitis, emphysema or pneumonia, or for any (Table 7). A total of 22% of case subjects and 27% of control subjects reported a physician-diagnosed nonmalignant lung disease, for an odds ratio of 0.9 (95% CI = 0.5–1.7). A positive family history of cancer in first-degree relatives demonstrated no increased lung-cancer risk (OR = 0.7, 95% CI = 0.4–1.3). The same holds true for a positive family history of lung cancer. Again there was no major influence on the results of previous lung disease or family history after additional adjustment for other potential risk factors.

### DISCUSSION

This study aimed to explore the contribution of various potential risk factors to lung cancer among male nonsmokers. This is presently one of the very few studies focusing exclusively on nonsmoking men. However, numbers of case subjects were limited. As in several other studies in nonsmoking lung cancer patients adenocarcinoma was the predominant cell subtype (59%). Our findings suggest that exposure to occupational carcinogens and elevated radon concentrations in the home appear to be risk factors for lung cancer in nonsmoking men. In addition there is weak evidence that ETS exposure, particularly outside the home as in transportation and at social settings, may play a role.

### Occupation

Several occupational exposures such as asbestos, arsenic, chromium, cadmium, and nickel are well established as lung carcinogens among men and smokers (Samet, 1994). The few available studies in nonsmoking men also suggest that certain occupations may contribute to lung cancer (Pohlabeln et al, 2000; Keller and Howe, 1993). A study in Illinois showed that white male nonsmoking cases were significantly more likely to have worked in the construction industry, in the bus/trucking service

**Table 6** Odds ratio of lung cancer and cumulative duration of exposure to various sources of environmental tobacco smoke

| Source ETS-exposure | Cases (*n*) | Controls (*n*) | OR | 95% CI |
|---|---|---|---|---|
| Spouse | | | | |
| no | 53 | 724 | 1.0 | Referent |
| low | 3 | 53 | 0.8 | 0.23–2.65 |
| high | 1 | 18 | 0.8 | 0.11–6.38 |
| Workplace | | | | |
| no | 20 | 233 | 1.0 | Referent |
| low | 18 | 271 | 0.8 | 0.39–1.48 |
| high | 8 | 89 | 1.1 | 0.47–2.70 |
| Transportation | | | | |
| no | 47 | 697 | 1.0 | Referent |
| low | 6 | 77 | 1.2 | 0.51–3.02 |
| high | 4 | 24 | 2.7 | 0.88–8.03 |
| Social settings | | | | |
| no | 30 | 488 | 1.0 | Referent |
| low | 20 | 215 | 1.6 | 0.87–2.90 |
| high | 8 | 87 | 1.5 | 0.67–3.44 |
| Summary indicator outside the home | | | | |
| no | 10 | 150 | 1.0 | Referent |
| low | 23 | 287 | 1.2 | 0.53–2.54 |
| high | 13 | 145 | 1.4 | 0.59–8.41 |

Missing values were excluded for each source of environmental tobacco smoke exposure; OR = odds ratio adjusted for age and region.

© 2001 Cancer Research Campaign

**Table 7** Odds ratios for lung cancer and previous lung disease (diagnosed by a physician at least 2 years before interview) and family history of cancer (first-degree relatives)

| | Positive/negative history | | | |
|---|---|---|---|---|
| | Cases (*n*) | Controls (*n*) | OR | 95% CI |
| Previous lung disease | | | | |
| Tuberculosis | 3/55 | 35/764 | 1.2 | 0.04–1.41 |
| Asthma | 2/56 | 27/776 | 1.1 | 0.26–4.82 |
| Chronic bronchitis | 4/54 | 75/726 | 0.8 | 0.29–2.35 |
| Emphysema | 0/58 | 11/792 | – | – |
| Pneumonia | 6/51 | 131/667 | 0.6 | 0.26–1.49 |
| Any lung disease | 13/45 | 213/590 | 0.9 | 0.45–1.65 |
| Family history of | | | | |
| Cancer | 14/44 | 265/538 | 0.7 | 0.36–1.25 |
| Lung cancer | 2/56 | 36/767 | 0.8 | 0.19–3.46 |

OR = odds ratio adjusted for age and area.

industry, and in blast furnaces, steelworks and rolling and finishing mills than controls (Keller and Howe, 1993). A recent European multicentre case–control study in male nonsmokers, including 39 cases and 79 controls from the present study, found a non-significantly raised risk (OR = 1.5, 95% CI = 0.8–3.0) for exposure to known occupational lung carcinogens (A-list), but no trend in risk with respect to duration of employment in A- or B-list jobs (Pohlabeln et al, 2000). In contrast, we found an increase in risk with increasing duration, and higher odds ratios. These differences in risk, however, and also those between East and West Germany may either be random or may reflect differences in the intensity or prevalence of lung carcinogens summarized by each job title by country. We believe that the observed strong effect of occupation is unlikely to be explained by confounding bias and also recall bias should be small (McGuire et al, 1998).

### Radon

While earlier studies have provided inconsistent results, there is growing evidence from well-performed large-sized case–control studies that residential radon is associated with a small but consistent increased lung-cancer risk. The totality of the evidence from these studies suggests an ERR of about 0.10 per 100 Bq $m^{-3}$ (Pershagen et al, 1994; Auvinen et al, 1996; Lubin and Boice, 1997; Darby et al, 1998; Kreienbrock et al, 2000). A recent review of radon studies with risk estimates for nonsmokers (Brownson et al, 1998) indicated rather inconsistent results. Three case–control studies in nonsmoking women in Missouri (Alavanja et al, 1994), China (Blot et al, 1990) and New Jersey (Schoenberg et al, 1990) found no positive trend in risk, in contrast to a Stockholm study of women (Pershagen et al, 1992), where lung-cancer risk tended to increase with increasing radon exposure for both never-smokers and current smokers.

Overall, we found an unexpectedly high ERR of 0.43, which differed between the eastern (ERR = 0.60, $P < 0.05$) and the western (ERR = –0.74, n.s.) study area. As in other small studies of nonsmokers our results require cautious interpretation. In the western study areas there were no high radon concentrations in homes, so the range of values was rather small, and with the small numbers of subjects, the power to detect any effect was limited. Inaccuracy of measurements is an additional important methodological problem. It has been suggested that paticularly in studies with low radon concentrations inaccuracy of measurements tends to dilute an effect. To dilute an effect (Darby et al, 1998; Kreienbrock et al, 2000). On the other hand, there was a clear association between radon and lung cancer in the eastern study area, where many case and control subjects lived in houses with high radon concentrations and all risk estimates were statistically significant, although with broad confidence intervals. It seems unlikely that such a great effect is totally produced by random or potential bias. A confounding effect due to occupation, environmental tobacco smoke or diet at least was not observed.

Although analyses stratified by histologic type were hampered by small numbers, it was remarkable that on average patients with small cell lung cancer showed 2.3-fold higher radon concentrations in their homes than non-small cell lung cancer patients. This finding is consistent with those of other indoor radon or uranium miner studies, suggesting that small cell lung cancers are more likely to be induced by radiation than others (BEIR VI, 1998).

### Environmental tobacco smoke

In agreement with other studies (Kabat et al, 1995; Boffetta et al, 1998; Kreuzer et al, 2000), the most frequently reported source of exposure to ETS in adulthood was not at home (as it is for women), but rather outside the home. No effect due to ETS exposure from spouses was observed, although the numbers were small. A joint analysis of nine studies including a total of 274 male lung cancer patients (Hackshaw et al, 1997) showed a 1.34-fold relative risk (95% CI = 0.97–1.84) for nonsmoking men whose spouses smoked, as compared to those whose spouses did not smoke. Our study indicated no elevated lung cancer risk due to ETS exposure at work and slightly elevated risk estimates associated with high ETS exposure during transportation or in social settings, but confidence intervals were broad. These findings add to the evidence of other studies (Kabat et al, 1995; Boffetta et al, 1998).

### Other risk factors

In the control population, the prevalence of previous tuberculosis (4.4%), asthma (3.4%) and emphysema (1.4%) was relatively low, while that of chronic bronchitis (9.4%) and pneumonia (16.4%) was somewhat higher. These proportions are in the range reported in other studies on nonsmoking men and women (Mayne et al, 1999; Wu et al, 1995; Alavanja et al, 1992). Yet these studies have provided evidence that certain previous nonmalignant lung diseases may be a risk factor for lung cancer; our lack of such

© 2001 Cancer Research Campaign evidence may simply be due to the small power. Schwartz et al (1996) reported a positive family history of lung cancer as strong risk factor for lung cancer in nonsmokers only in the group of early-onset cases (40–59 years of age) (OR = 7.2, 95% CI = 1.3–39.7) and not in the older age group (OR = 1.1, 95% CI = 0.6–2.1). Such an age-dependency of familial aggregation of lung cancer was confirmed by other studies (Kreuzer et al, 1998; Gauderman and Morrison, 2000). Since a genetic predisposition seems to be linked only to very young lung cancer cases, it was not surprising that in the present analysis over all ages no effect was detected.

### Strengths and weaknesses

The major strengths of our investigation are that all patients were interviewed in person and no data were obtained by next-of-kin or other surrogates. Detailed information on the main risk factors such as occupation, environmental tobacco smoke, previous lung disease and family history of cancer was ascertained by closely supervised, trained interviewers and standardized questionnaires. For most study subjects, 1-year radon measurements in their current homes were available. Finally, all cases had histologically or cytologically confirmed, primary lung tumors, and histological subtypes were evaluated by one reference pathologist.

Nevertheless, there are several limitations in our study concerning potential bias due to the selection of case and control subjects, misclassification of nonsmoking status, uncertainty in radon measurements and instability of risk estimates based on the small numbers of case subjects. Some methodological problems concern the low response rates among control subjects. Unfortunately, response rates cannot be provided seperately for smokers and non-smokers, since this information was not gathered. The low response rate among population controls was mainly due to the refusal of long-term measurements of radon in their homes (Kreuzer et al, 1998). Detailed analysis of a subsample of non-responders had demonstrated that better educated people tended to be more willing to participate (Kreienbrock et al, 2000). Since in Germany a higher educational level is associated with a higher proportion of nonsmokers in men, response rates should be higher for nonsmokers than overall. The effect of selection bias cannot be completely ruled out. Additional adjustment for educational level, however, did not change the results on occupation and residential radon. The recruitment of lung cancer patients which in Germany has to be done via hospitals, since no overall cancer registries exist is another concern of potential bias, thus the representativeness of our case subjects to all cases is not measurable. In previous analyses based on the former East German cancer registry we estimated a coverage of about 50% for the eastern study area.

Another source of possible bias, especially relevant to the effects of ETS, is misclassification of ever-smokers as lifetime never-smokers. Smoking status was not validated in our study. Results of a European validation study using cross-interview with next of kin suggest that bias from nonsmoker misclassification is not likely to be significant (Nyberg et al, 1998).

In summary, our study suggests that occupation and residential radon may be important risk factors for lung cancer in nonsmoking men. In addition, exposure to environmental tobacco smoke outside the home may have some weak influence. These findings indicate that among nonsmoking men other factors may contribute to lung-cancer risk than among nonsmoking women. Our study is limited by small numbers of male cases, so larger studies or pooled analyses of studies on nonsmoking men are needed to confirm these findings.

### REFERENCES

Ahrens W and Merletti F (1998) A standard tool for the analysis of occupational lung cancer in epidemiologic studies. *Int J Occup Environ Health* **4**: 236–240

Alavanja MCR, Brownson RC, Boice JD and Hock E (1992) Preexisting lung disease and lung cancer among nonsmoking women. *Am J Epidemiol* **136**: 623–632

Alavanja MCR, Brownson RC, Lubin JH, Berger E, Chang J and Boice JD (1994) Residential radon exposure and lung cancer among nonsmoking women. *J Natl Cancer Inst* **86**: 1829–1837

Auvinen A, Mäkeläinen I, Hakama M, Castren O, Pukkala E, Reisbacka H and Rytömaa T (1996) Indoor radon exposure and risk of lung cancer: a nested case-control study in Finland. *J Natl Cancer Inst* **88**: 966–972 (Erratum: *J Natl Cancer Inst* 1998; **90**: 401–402)

BEIR VI (Committee on biological effects of ionizing radiation) (1998) *Health effects of exposure to radon*. Academy Press: Washington DC

Bennett WP, Alavanja MCR, Blomeke B, Vähäkangas KH, Castren K, Welsh JA, Bowman ED, Khan MA, Flieder DB and Harris CC (1999) Environmental tobacco smoke, genetic susceptibility, and risk of lung cancer in never-smoking women. *J Natl Cancer Inst* **91**: 2009–2014

Blot WJ, Xu Z-Y, Boice JD, Zhao D-Z, Stone BJ, Sun J, Jing LB and Fraumeni JF Jr (1990) Indoor radon and lung cancer in China. *J Natl Cancer Inst* **82**: 1025–1030

Boffetta P, Agudo A, Ahrens W, Benhamou S, Benhamou E, Darby S, Ferro G, Fortes C, Gonzalez CA, Jöckel KH, Krauss M, Kreienbrock L, Kreuzer M, Mendez A, Merletti F, Nyberg F, Pershagen G, Pohlabeln H, Riboli E, Schmid G, Simonato L, Tredaniel J, Whitley E, Wichmann HE, Winck C, Zambon P and Saracci R (1998) Multicentre case-controls study of exposure to environmental tobacco smoke and lung cancer in Europe. *J Natl Cancer Inst* **90**: 1440–1450

Brennan P, Fortes C, Butler J, Agudo A, Benhamou S, Darby S, Gerken M, Jöckel KH, Kreuzer M, Mallone S, Nyberg F, Pohlabeln H, Ferro G and Boffetta P (2000) A multicentre case-control study of diet and lung cancer among nonsmokers. *Cancer Causes Control* **11**: 49–58

Breslow NE and Day NE (1980) *Statistical methods in cancer research*, Vol 1. The analysis of case-control studies. International Agency for Research on Cancer Lyon.

Brownson RC, Alavanja MCR, Caporaso N, Berger E and Chang JC (1997) Family history of lung cancer in lifetime non-smokers and long-term exsmokers. *Int J Epidemiol* **26**: 256–263

Brownson RC, Alavanja MCR, Caporaso N, Simoes EJ and Chang JC (1998) Epidemiology and prevention of lung cancer in nonsmokers. *Epidemiol Reviews* **20**: 218–236

Brüske-Hohlfeld I, Möhner M, Pohlabeln H, Ahrens W, Bolm-Audorff U, Kreienbrock L, Kreuzer M, Jahn I, Wichmann HE and Jöckel KH (2000) Occupational lung cancer risk for men in Germany: results of a pooled case-control study. *Am J Epidemiol* **151**: 384–395

Darby SC, Whitley E, Silcocks P, Tharkar B, Green M, Lomas P, Miles J, Reeves G, Fearn T and Doll R (1998) Risk of lung cancer associated with residential radon exposure in south-west England: a case-control study. *Br J Cancer* **78**: 394–408

Gauderman WJ and Morrison JL (2000) Evidence for age-specific genetic relative risks in lung cancer. *Am J Epidemiol* **151**: 41–49

Gerken M, Kreienbrock L, Wellmann J, Kreuzer M and Wichmann HE (2000) Models for retrospective quantification of indoor radon exposure in case-control studies. *Health Phys* **78**: 268–278

Hackshaw AK, Law MR and Wald NJ (1997) The accumulated evidence on lung cancer and environmental tobacco smoke. *BMJ* **315**: 980–988

Kabat GC, Stellman SD and Wynder EL (1995) Relation between exposure to environmental tobacco smoke and lung cancer in lifetime nonsmokers. *Am J Epidemiol* **142**: 141–148

Keller JE and Howe HL (1993) Risk factors for lung cancer among nonsmoking Illinois residents. *Environ Res* **60**: 1–11

Koo LC and Ho JHC (1990) Worldwide epidemiological patterns of lung cancer in nonsmokers. *Int J Epidemiol* **19** (Suppl 1): S14–23

Kreienbrock L, Kreuzer M, Gerken M, Dingerkus G, Wellmann J, Keller G and Wichmann HE (2000) Case-control study on lung cancer and residential radon in West Germany. *Am J Epidemiol*: in press

Kreuzer M, Kreienbrock L, Gerken M, Heinrich J, Brüske-Hohlfeld I, Müller KH and Wichmann HE (1998) Risk factors for lung cancer in young adults. *Am J Epidemiol* **147**: 1028–1037

*© 2001 Cancer Research Campaign* 

Kreuzer M, Pohlabeln H, Ahrens W, Kreienbrock L, Brüske-Hohlfeld I, Jöckel KH and Wichmann HE (1999) Occupational risk factors for lung cancer in young males. *Scand J Work Environ Health* **25**: 423–430
Kreuzer M, Krauss M, Kreienbrock L, Jöckel KH and Wichmann HE (2000) Environmental tobacco smoke and lung cancer: a case-control study in Germany. *Am J Epidemiol* **151**: 241–250
Lubin JH and Boice JD (1997) Lung cancer risk from residential radon: meta-analysis of eight epidemiologic studies. *J Natl Cancer Inst* **89**: 49–57
Mayne ST, Buenconsejo J and Janerich DT (1999) Previous lung disease and risk of lung cancer among men and women nonsmokers. *Am J Epidemiol* **149**: 13–20
McGuire V, Nelson LM, Koepsell TD, Checkoway H and Longstreth WT (1998) Assessment of occupational exposures in community-based case-control studies. *Ann Rev Public Health* **19**: 35–53
Nyberg F, Agudo A, Boffetta P, Fortes C, Conzalez CA and Pershagen G (1998) A European validation study of smoking and environmental tobacco smoke exposure in nonsmoking lung cancer cases and controls. *Cancer Causes Control* **9**: 173–182
Parkin DM, Pisani P, Lopez AD and Masuyer E (1994) At least one in seven cases of cancer is caused by smoking. Global estimates for 1985. *Int J Cancer* **59**: 494–504
Pershagen G, Liang ZH, Hrubec Z, Svensson C and Boice JD (1992) Residential radon exposure and lung cancer in women. *Health Phys* **63**: 179–186
Pershagen G, Akerblom G, Axelson O, Clavensjö B, Damber L, Desai G, Enflo A, Lagarde F, Mellander H, Svartengren M and Swedjemark GA (1994) Residential radon exposure and lung cancer in Sweden. *N Engl J Med* **330**: 159–164
Pohlabeln H, Boffetta P, Ahrens W, Merletti F, Agudo A, Benhamou E, Benhamou S, Brueske-Hohlfeld I, Ferro G, Fortes C, Kreuzer M, Mendes A, Nyberg F, Pershagen G, Saracci R, Schmid G, Siemiatycki J, Simonato L, Whitley E, Wichmann HE, Winck C and Jöckel KH (2000) Occupational risks for lung cancer: results from a multicentre case-control study in nonsmokers. *Epidemiology* **11**: 532–538
Samet JM (1994) Epidemiology of lung cancer. Marcel Dekker, Inc.: New York
Schoenberg JB, Klotz JB, Wilcox HB, Nicholls GP, Gil-de-Real MT, Stemhagen A and Mason TJ (1990) Case-control study of residential radon and lung cancer among New Jersey women. *Cancer Res* **50**: 6250–6254
Schwartz AG, Yang P and Swanson GM (1996) Familial risk of lung cancer among nosmokers and their relatives. *Am J Epidemiol* **144**: 554–562
Statistisches Bundesamt (1975). Klassifizierung der Berufe: Systematisches und alphabetisches Verzeichnis der Berufsnennung. Verlag W Kohlhammer: Stuttgart/Mainz
Wu AH, Fontham ETH, Reynolds P, Greenberg RS, Buffler P, Liff J, Boyd P, Henderson BE and Correa P (1995) Previous lung disease and risk of lung cancer among lifetime nonsmoking women in the United States. *Am J Epidemiol* **141**: 1023–1032

 *© 2001 Cancer Research Campaign*

## References

[PDF_00595] Ahrens W., Merletti F. (1998). A standard tool for the analysis of occupational lung cancer in epidemiologic studies.. Int J Occup Environ Health.

[PDF_00597] Alavanja M. C., Brownson R. C., Boice J. D., Hock E. (1992). Preexisting lung disease and lung cancer among nonsmoking women.. Am J Epidemiol.

[PDF_00599] Alavanja M. C., Brownson R. C., Lubin J. H., Berger E., Chang J., Boice J. D. (1994). Residential radon exposure and lung cancer among nonsmoking women.. J Natl Cancer Inst.

[PDF_00602] Auvinen A., Mäkeläinen I., Hakama M., Castrén O., Pukkala E., Reisbacka H., Rytömaa T. (1996). Indoor radon exposure and risk of lung cancer: a nested case-control study in Finland.. J Natl Cancer Inst.

[PDF_00608] Bennett W. P., Alavanja M. C., Blomeke B., Vähäkangas K. H., Castrén K., Welsh J. A., Bowman E. D., Khan M. A., Flieder D. B., Harris C. C. (1999). Environmental tobacco smoke, genetic susceptibility, and risk of lung cancer in never-smoking women.. J Natl Cancer Inst.

[PDF_00612] Blot W. J., Xu Z. Y., Boice J. D., Zhao D. Z., Stone B. J., Sun J., Jing L. B., Fraumeni J. F. (1990). Indoor radon and lung cancer in China.. J Natl Cancer Inst.

[PDF_00615] Boffetta P., Agudo A., Ahrens W., Benhamou E., Benhamou S., Darby S. C., Ferro G., Fortes C., Gonzalez C. A., Jöckel K. H. (1998). Multicenter case-control study of exposure to environmental tobacco smoke and lung cancer in Europe.. J Natl Cancer Inst.

[PDF_00622] Brennan P., Fortes C., Butler J., Agudo A., Benhamou S., Darby S., Gerken M., Jökel K. H., Kreuzer M., Mallone S. (2000). A multicenter case-control study of diet and lung cancer among non-smokers.. Cancer Causes Control.

[PDF_00629] Brownson R. C., Alavanja M. C., Caporaso N., Berger E., Chang J. C. (1997). Family history of cancer and risk of lung cancer in lifetime non-smokers and long-term ex-smokers.. Int J Epidemiol.

[PDF_00632] Brownson R. C., Alavanja M. C., Caporaso N., Simoes E. J., Chang J. C. (1998). Epidemiology and prevention of lung cancer in nonsmokers.. Epidemiol Rev.

[PDF_00635] Brüske-Hohlfeld I., Möhner M., Pohlabeln H., Ahrens W., Bolm-Audorff U., Kreienbrock L., Kreuzer M., Jahn I., Wichmann H. E., Jöckel K. H. (2000). Occupational lung cancer risk for men in Germany: results from a pooled case-control study.. Am J Epidemiol.

[PDF_00639] Darby S., Whitley E., Silcocks P., Thakrar B., Green M., Lomas P., Miles J., Reeves G., Fearn T., Doll R. (1998). Risk of lung cancer associated with residential radon exposure in south-west England: a case-control study.. Br J Cancer.

[PDF_00642] Gauderman W. J., Morrison J. L. (2000). Evidence for age-specific genetic relative risks in lung cancer.. Am J Epidemiol.

[PDF_00644] Gerken M., Kreienbrock L., Wellmann J., Kreuzer M., Wichmann H. E. (2000). Models for retrospective quantification of indoor radon exposure in case-control studies.. Health Phys.

[PDF_00647] Hackshaw A. K., Law M. R., Wald N. J. (1997). The accumulated evidence on lung cancer and environmental tobacco smoke.. BMJ.

[PDF_00649] Kabat G. C., Stellman S. D., Wynder E. L. (1995). Relation between exposure to environmental tobacco smoke and lung cancer in lifetime nonsmokers.. Am J Epidemiol.

[PDF_00652] Keller J. E., Howe H. L. (1993). Risk factors for lung cancer among nonsmoking Illinois residents.. Environ Res.

[PDF_00654] Koo L. C., Ho J. H. (1990). Worldwide epidemiological patterns of lung cancer in nonsmokers.. Int J Epidemiol.

[PDF_00667] Kreuzer M., Krauss M., Kreienbrock L., Jöckel K. H., Wichmann H. E. (2000). Environmental tobacco smoke and lung cancer: a case-control study in Germany.. Am J Epidemiol.

[PDF_00656] Kreuzer M., Kreienbrock L., Gerken M., Heinrich J., Bruske-Hohlfeld I., Muller K. M., Wichmann H. E. (1998). Risk factors for lung cancer in young adults.. Am J Epidemiol.

[PDF_00670] Lubin J. H., Boice J. D. (1997). Lung cancer risk from residential radon: meta-analysis of eight epidemiologic studies.. J Natl Cancer Inst.

[PDF_00672] Mayne S. T., Buenconsejo J., Janerich D. T. (1999). Previous lung disease and risk of lung cancer among men and women nonsmokers.. Am J Epidemiol.

[PDF_00674] McGuire V., Nelson L. M., Koepsell T. D., Checkoway H., Longstreth W. T. (1998). Assessment of occupational exposures in community-based case-control studies.. Annu Rev Public Health.

[PDF_00677] Nyberg F., Agudo A., Boffetta P., Fortes C., González C. A., Pershagen G. (1998). A European validation study of smoking and environmental tobacco smoke exposure in nonsmoking lung cancer cases and controls.. Cancer Causes Control.

[PDF_00681] Parkin D. M., Pisani P., Lopez A. D., Masuyer E. (1994). At least one in seven cases of cancer is caused by smoking. Global estimates for 1985.. Int J Cancer.

[PDF_00685] Pershagen G., Akerblom G., Axelson O., Clavensjö B., Damber L., Desai G., Enflo A., Lagarde F., Mellander H., Svartengren M. (1994). Residential radon exposure and lung cancer in Sweden.. N Engl J Med.

[PDF_00683] Pershagen G., Liang Z. H., Hrubec Z., Svensson C., Boice J. D. (1992). Residential radon exposure and lung cancer in Swedish women.. Health Phys.

[PDF_00689] Pohlabeln H., Boffetta P., Ahrens W., Merletti F., Agudo A., Benhamou E., Benhamou S., Brüske-Hohlfeld I., Ferro G., Fortes C. (2000). Occupational risks for lung cancer among nonsmokers.. Epidemiology.

[PDF_00700] Schwartz A. G., Yang P., Swanson G. M. (1996). Familial risk of lung cancer among nonsmokers and their relatives.. Am J Epidemiol.

[PDF_00705] Wu A. H., Fontham E. T., Reynolds P., Greenberg R. S., Buffler P., Liff J., Boyd P., Henderson B. E., Correa P. (1995). Previous lung disease and risk of lung cancer among lifetime nonsmoking women in the United States.. Am J Epidemiol.

